# Abdominal compartment syndrome during hip arthroscopy for an acetabular fracture: a case report

**DOI:** 10.1186/s40981-017-0100-y

**Published:** 2017-05-08

**Authors:** Tomoharu Shakuo, Kiyoko Bito, Seiichi Yasuda, Chie Asagi

**Affiliations:** 1 Department of Anesthesiology, Showa University Northern Yokohama Hospital, 35-1 Chigasaki Chuou, Tsuzuki-ku, Yokohama-shi, Kanagawa 224-8503 Japan; 2 Department of Anesthesiology, Showa University, 1-5-8 Hatanodai, Shinagawa-ku, Tokyo, 142-8555 Japan; 3 Nissan Tamagawa Hospital, 4-8-1 Seta, Setagaya-ku, Tokyo, 158-0095 Japan

**Keywords:** Abdominal compartment syndrome, Hip arthroscopy, Fluid extravasation

## Abstract

**Background:**

We encountered a case of abdominal compartment syndrome during hip arthroscopic surgery, caused by the irrigation fluid flowing into the peritoneal cavity.

**Case presentation:**

A 47-year-old male patient with the acetabulum fracture underwent open reduction and internal fixation with hip arthroscopy. Hypothermia, increased airway pressure (under volume-controlled ventilation) and oliguria were observed during the operation, and arterial blood gas analysis showed decreased oxygenation and metabolic acidosis. Abdominal distention was observed, and a postoperative CT revealed accumulation of a large volume of irrigation fluid in the peritoneal cavity and retroperitoneum. The patient was diagnosed as having abdominal compartment syndrome and treated by percutaneous peritoneal drainage. His subsequent course was uneventful, and he was discharged 8 weeks after the operation. Intraperitoneal extravasation of irrigation fluid may occur during hip arthroscopic surgery, and is more likely to occur in the presence of an injury.

**Conclusion:**

Anesthesiologists should be aware of the possible occurrence of the abdominal compartment syndrome during hip arthroscopic surgery and ensure that it is detected early.

## Background

Recently, hip arthroscopic surgery has been increasingly performed, because it requires a smaller surgical wound, is less invasive, and enables early postoperative return of the patients to their daily life as compared to open surgery. During this surgery, irrigation fluid is injected into the hip joint to expand it, and the operation is performed under magnifying-endoscopic observation. Although complications have been reported to occur in less than 1.5% [1] and symptomatic intraperitoneal accumulation of irrigation fluid is extremely rare (0.16% incidence) [2], a recent study with postoperative ultrasound examination revealed that 16% of patients have intraabdominal fluid extravasation after hip arthroscopy [3]. Herein, we report a case in which the injected irrigation fluid during hip arthroscopic surgery accumulated in the peritoneal cavity and retroperitoneum, causing abdominal compartment syndrome.

## Case presentation

**Fig. 1:**
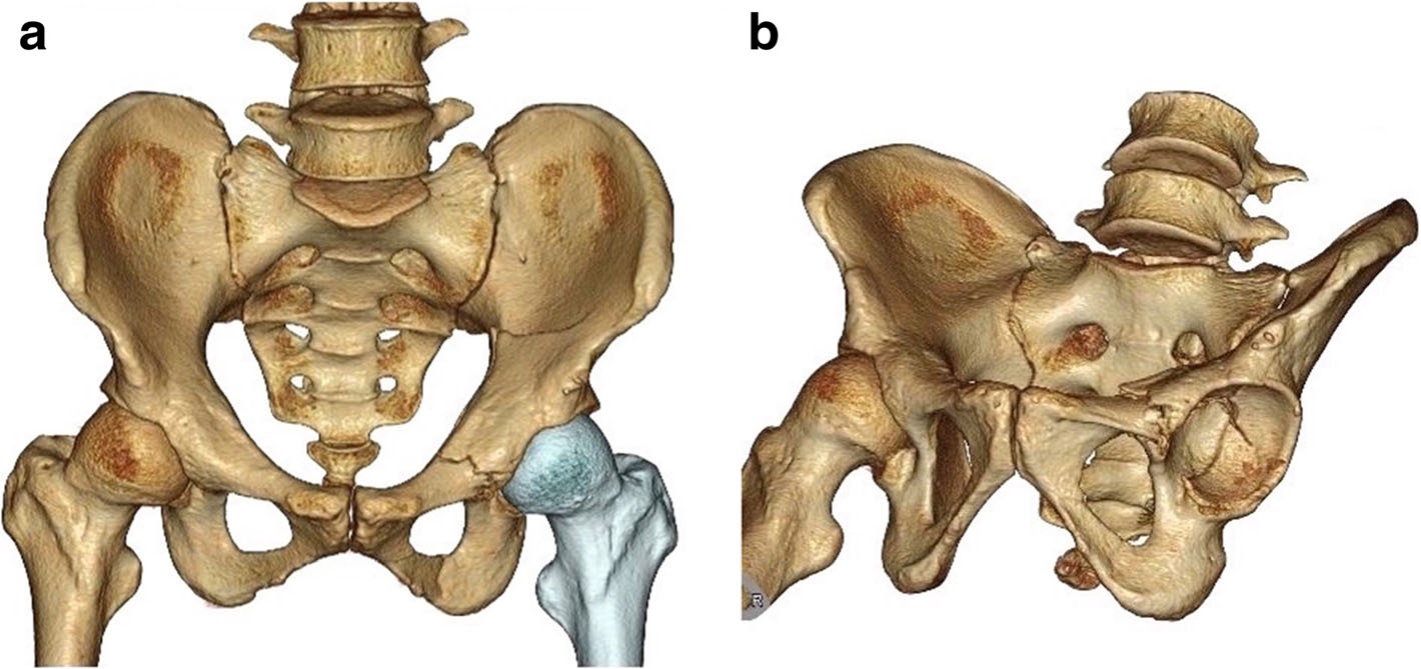
Preoperative 3D-CT. **a** Frontal view. **b** Left oblique view. A fracture of the left innominate bone and a fracture of the left acetabulum are observed

**Fig. 2:**
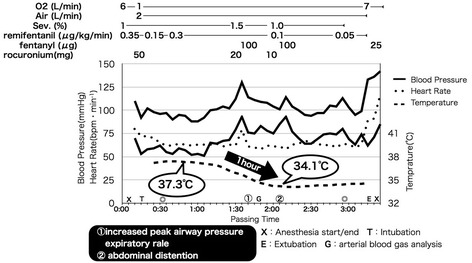
Anesthetic course

**Fig. 3:**
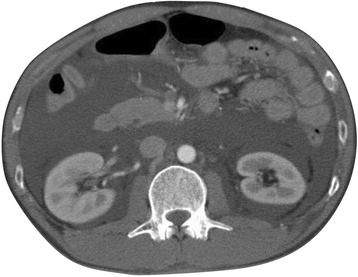
Postoperative abdominal CT revealed fluid accumulation in the peritoneal cavity and retroperitoneum

The patient was a 47-year-old male patient with a height of 175 cm and body weight of 59 kg, with no significant past medical or family history. He sustained injury when he hit against a tree while snowboarding. Radiography and CT at our hospital revealed no obvious abdominal organ injuries, but a fracture of the left acetabulum, and open reduction and internal fixation under arthroscopic guidance was planned (Fig. 1). General anesthesia was rapidly induced with propofol, rocuronium and remifentanil, and maintained with sevoflurane (Fig. 2). Hip traction was performed using a traction table in the supine position. An anterolateral portal and a mid-anterior portal was used. Osteosynthesis was performed using the pinning technique under fluoroscopic guidance, and fracture sight was confirmed by hip arthroscopy. Forty minutes after start of the operation, the surgeons noted remarkable bleeding through arthroscopy and demanded to increase the peak perfusion to 100 mmHg in order to secure their visual field. Hemodynamics parameters such as blood pressure and heart rates remained stable. However, the esophageal temperature began to decrease, from 37.3 to as low as 34.1 °C. Ninety-five minutes after start of the operation, the peak airway pressure in volume-controlled ventilation increased from 18 to 25 cm H_2_O without alteration in end-tidal CO2 curves. Additional rocuronium and tracheal aspiration couldn’t make the patient’s airway pressure reduce. No hemodynamic abnormalities were observed, however, there was no urine output. The arthroscopy was completed in approximately 2 h. Abdominal examination revealed distention. The 24,000 mL of irrigation fluid (lactated ringers: each liter contains 6.0 g sodium chloride, USP, 3.1 g sodium lactate, 300 mg potassium chloride and 200 mg calcium chloride) injected into the joint during arthroscopy, but the 1,300 mL or more of them failed to be retrieved. Arterial blood gas analysis at this time revealed decreased oxygenation and metabolic acidosis under an FiO_2_ of 0.4: PaO_2_ 72.0 mm Hg, PaCO_2_ 39.6 mm Hg, pH 7.323, HCO_3_^−^ 20.5 and BE −5.0. The total operation time was 2 h 23 min. The patient emerged quite soon from the anesthesia, but became restless after extubation. Abdominal CT was performed urgently, which revealed accumulation of a large volume of fluid that seemed to be the irrigation fluid in the peritoneal cavity and retroperitoneum (Fig. 3). One hour after the operation, arterial blood gas analysis under oxygen supplementation at 10 L/min via a face oxygen mask revealed marked worsening of the metabolic acidosis: PaO_2_ 105.9 mm Hg, PaCO_2_ 43.2 mm Hg, pH 7.082, HCO_3_^−^ 12.3 and BE −15.2. A chest X-ray showed pulmonary congestion with diaphragmatic elevation, but there was no apparent fluid accumulation in the pleural cavity. The patient was diagnosed clinically as having abdominal compartment syndrome, and percutaneous peritoneal drainage was performed. About 800 mL of bloody or slightly bloody fluid was drained. Immediately after the drainage, the patient’s level of consciousness and oxygenation improved dramatically, and the oxygen supplementation was withdrawn on postoperative day 1. There were no electrolyte disorders during the perioperative period. The postoperative pain delayed the patient’s recovery. Although the patient was started on “bedridden rehabilitation” 2 days after the operation, it took 4 weeks before he could be started on gait training. He was discharged 8 weeks after the operation.

### Discussion

From the present case, the following two points are noteworthy: abdominal compartment syndrome can occur due to the accumulation of irrigation fluid during hip arthroscopic surgery, and percutaneous peritoneal drainage is effective for treating this complication.

Takagi et al. performed hip arthroscopy for the first time in the world in 1935, and Aignan et al. successfully performed hip arthroscopic surgery in 1976 [4]. This surgery is indicated for acetabular labral tears and femoroacetabular impingement (FAI). Recently, basic techniques of hip arthroscopic surgery have been established and the safety of this surgery has improved. However, various complications still occur; the major complications are neuropathy and skin disorders due to body position, iatrogenic injury due to the surgical procedures (glenoid labrum and cartilage), hypothermia due to the use of irrigation fluid, and intraperitoneal extravasation of the irrigation fluid [5].

The mechanism underlying the intraperitoneal extravasation of the irrigation fluid still remains unclear. It has been reported that the irrigation fluid may flow along the iliopsoas muscle and external iliac artery and vein, reach the retroperitoneum, and enter the peritoneal cavity through congenital communications between the retroperitoneum and peritoneal cavity [6]. In addition, Bartlett et al. speculated that in trauma cases, peritoneal damage results in communications opening up between the retroperitoneum and the peritoneal cavity, causing the entry of irrigation fluid into the peritoneal cavity [7]. Furthermore, Kocher et al. reported that a high perfusion pressure is a risk factor for the development of this complication [8]. In the present case, peritoneal damage at the time of the accident was suspected, which, in addition to the high perfusion pressure, could have caused the intraperitoneal extravasation of the irrigation fluid. Sudden hypothermia has also been pointed out as an important finding suggestive of intraperitoneal extravasation of the irrigation fluid [6, 7, 9].

Abdominal compartment syndrome during hip arthroscopy is a rare, but serious, complication. In fact, there is even a case report of cardiac arrest caused by this complication [7]. Abdominal compartment syndrome is a condition characterized by sustained increase of the abdominal pressure (20 mm Hg or higher), leading to new organ dysfunction [10]. The pathophysiological outcomes include blood flow decrease to the intraperitoneal and retroperitoneal organs, reduced venous return to the heart and increased peripheral vascular resistance due to vascular compression with a resultant decrease of the cardiac output, oliguria due to the compression of the renal parenchyma and renal veins, and respiratory insufficiency due to diaphragmatic elevation. Its progression is associated with multiple organ failure, including circulatory shock, respiratory failure, renal failure and intestinal ischemia. According to the algorithm of the World Society of the Abdominal Compartment Syndrome, treatment must be initiated in cases where the abdominal pressure is 12 mm Hg or higher. Trans-bladder pressure can be used and recommended instead of abdominal pressure due to its simplicity and low cost [10]. For treatment, improvement of the abdominal wall compliance through evacuation of the bowel and peritoneal contents, fluid management, improvement of organ perfusion and surgical decompression should be undertaken [10]. Percutaneous peritoneal drainage is an effective treatment, particularly in cases of intraperitoneal fluid accumulation. In the present case, there was clear evidence of organ dysfunction, such as oliguria, decreased oxygenation and progression of metabolic acidosis, in addition to intraperitoneal fluid accumulation observed on the CT images. During the operation, massive bleeding which required high perfusion pressure also increased the intra-abdominal pressure. The patient was diagnosed clinically as having abdominal compartment syndrome and treated by percutaneous drainage. As a result, rapid intra-abdominal decompression was achieved and the patient’s general condition improved markedly.

A recent study has suggested that extravasation of irrigation fluid after hip arthroscopy may be a common complication (16% incidence), and that it may be associated with postoperative pain [3]. This complication perhaps delays the patient’s recovery.

It is recommended that the abdominal pressure be measured every 4 h in patients with a risk of abdominal compartment syndrome [10]. In post-traumatic or long surgeries, it is necessary to consider monitoring the abdominal pressure. If increased abdominal pressure is noted, it is important to take early measures, including discontinuation of arthroscopy, for preventing the progression of organ dysfunction.

## Conclusions

Abdominal compartment syndrome may occur due to the accumulation of irrigation fluid during hip arthroscopic surgery. High perfusion pressures should be avoided, changes in the body temperature and airway pressure should be carefully monitored, and abdominal examination should be performed regularly. If any abnormality is noted, it is important to report it immediately to the operator and take appropriate measures. Percutaneous peritoneal drainage is a less invasive and effective treatment. Hip arthroscopic surgery is expected to become increasingly popular in the future, and anesthesiologists need to deepen their understanding of the many complications of this type of surgery and inform the patients of the possible risks of the procedure, including the risk of development of abdominal compartment syndrome.
